# Artificial intelligence-guided design of lipid nanoparticles for mRNA delivery

**DOI:** 10.1016/j.apsb.2025.11.029

**Published:** 2025-11-27

**Authors:** Kexin Su, Junjie Qiu, Tengfei Xu, Shuai Liu

**Affiliations:** a College of Pharmaceutical Sciences, State Key Laboratory of Advanced Drug Delivery and Release Systems, Zhejiang University, Hangzhou 310058, China; b Research Center for Clinical Pharmacy, College of Pharmaceutical Sciences, Zhejiang University, Hangzhou 310058, China; c Liangzhu Laboratory, Zhejiang University, Hangzhou 311121, China; d Eye Center of the Second Affiliated Hospital of Zhejiang University School of Medicine, Zhejiang University, Hangzhou 310009, China; e Zhejiang Key Laboratory of Pain Perception and Neuromodulation, Hangzhou 310009, China

**Keywords:** mRNA delivery, Lipid nanoparticles, Artificial intelligence, Machine learning, Deep learning, Nanomedicine, Precise delivery, mRNA therapeutics

## Abstract

Lipid nanoparticles (LNPs) hold significant potential for mRNA-based therapeutics, as evidenced by their successful use in SARS-CoV-2 mRNA vaccines. LNPs effectively protect and transport mRNA to target sites, thereby ensuring its stability and efficient transfection. Despite the progress, some challenges remain in the development of mRNA-LNP delivery systems, such as limited targeting specificity, the complexity of formulations, and the time-consuming and high-throughput screening process. Artificial intelligence (AI) has emerged as a powerful tool to address these challenges, accelerating the design and optimization process of LNPs. AI-guided approaches can improve the efficiency of lipid structure and formulation screening by rapidly identifying key design parameters and employing predictive modeling to optimize LNP properties. The combination of AI and LNP technology offers significant advantages, including enabling the design of more personalized and precise delivery systems, streamlining the development process, and reducing the cost. This review discusses recent advancements in AI-guided mRNA-LNP delivery systems and highlights their potential to revolutionize mRNA therapeutics.

## Graphical abstract

Artificial intelligence (AI)-guided approaches are revolutionizing lipid nanoparticle (LNP) mRNA delivery systems by optimizing design, predicting efficiency, and accelerating the development process, providing promising pathways to further optimize mRNA therapeutics.

**Figure:**
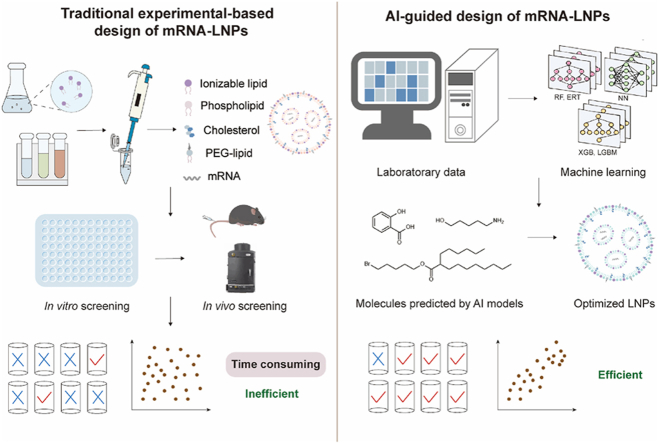

## 1 Introduction

Messenger RNA (mRNA)-based therapeutics have demonstrated substantial potential across various fields, including protein replacement therapy, gene editing, and diverse vaccines^1^, ^2^, ^3^. The unprecedented success of mRNA vaccines in combating the COVID-19 pandemic highlights the unique advantages of mRNA technology, such as rapid development, flexible design, and the capacity to elicit robust immune responses^4^, ^5^, ^6^, ^7^. These attributes have spurred a surge of interest in harnessing mRNA capabilities beyond vaccines, with promising applications to correct genetic defects, treat metabolic disorders, and develop personalized cancer vaccines^1^^,^^8^. As research and development continue to advance, mRNA therapeutics are anticipated to become the cornerstone of next-generation precision medicine, offering promising solutions for a wider range of diseases^2^^,^^9^^,^^10^. Despite these promising prospects, the clinical translation of mRNA therapeutics faces significant challenges, particularly in efficient delivery, stability, and immunogenicity. Recently, artificial intelligence (AI) has emerged as a transformative force in healthcare, revolutionizing fields such as drug discovery and nanomedicine^11^, ^12^, ^13^, ^14^. Especially, AI's capacity to analyze large data sets, predict molecular interactions, and optimize complex systems has opened new avenues for accelerating mRNA therapeutics^15^, ^16^, ^17^, ^18^.

Delivery issue represents the critical obstacle in mRNA therapeutic development^19^, ^20^, ^21^. Since utilized in FDA-approved two COVID-19 mRNA vaccines and one respiratory syncytial virus (RSV) mRNA vaccine, lipid nanoparticles (LNPs) have become the first choice for mRNA delivery^22^, ^23^, ^24^, ^25^, ^26^. Typically, LNPs consist of four key components: ionizable lipids, cholesterol, neutral phospholipids, and polyethylene glycol (PEG)-lipids^27^, ^28^, ^29^, synergistically aiding in mRNA encapsulation, cellular uptake, endosomal escape, and mRNA release into the cytoplasm^30^, ^31^, ^32^, ^33^. Despite the remarkable advancements in LNP technology, several challenges still remain, including rational design of ionizable lipids to enhance the delivery efficiency, optimizing LNP formulations to achieve tissue/cell-specificity, and minimizing off-target effects and immunogenicity. These challenges highlight the necessity for innovative tools to explore the vast compositional space of LNPs. With the rapid development of computer science, AI stands at the crossroads of multidisciplinary intersections specializing in all kinds of scientific issues related to data-intensity^34^, ^35^, ^36^, ^37^. AI has emerged as an aspirational tool in revolutionizing LNP development strategies and advancing mRNA therapeutics^4^^,^^38^, ^39^, ^40^. Relying on large amounts of high-quality training datasets, AI can extract insights from molecules and predict a wider range of lipid molecules based on their chemical structures and physicochemical properties^41^. By harnessing powerful data processing capabilities, AI offers the possibility to systematically analyze the sophisticated relationships among LNP compositions, chemical structural features, and functional effects. This facilitates high-throughput screening and lays the foundation for more efficient and personalized LNP design.

**Figure 1:**
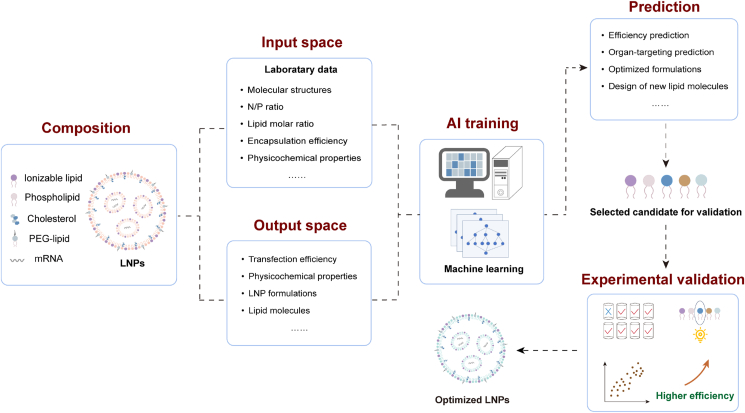
Overview of AI-guided design of LNPs.

The integration of AI into drug delivery research has already demonstrated its transformative impact, particularly through machine learning (ML) approaches^12^^,^^34^^,^^42^^,^^43^. These methods are beneficial to discovering concealed associations in large datasets, predicting formulation performances, and guiding the rational design of delivery systems. Applying AI to LNP design, researchers are able to explore expansive formulations with unprecedented efficiency, accelerating the discovery of novel lipid structures that enhance mRNA delivery while reducing reliance on labor-intensive and costly experimental iterations. Furthermore, AI-guided LNP design allows for a more comprehensive understanding of the multifaceted interactions between LNP components and biological systems, including factors such as biodistribution, cellular uptake mechanisms, and immune responses. The convergence of AI and nanotechnology holds substantial promise for overcoming existing limitations in mRNA delivery, enabling the development of LNPs with enhanced targeting capabilities, improved potency, and reduced adverse effects. With the continuous development, AI is expected to drive a paradigm shift in LNP engineering, providing new opportunities for mRNA therapeutics across a wide range of applications. This review will provide a comprehensive overview of the critical role of AI in guiding the design of LNPs for mRNA therapeutics (Fig. 1). By exploring recent advances in AI-guided LNP development, we focus on how data-driven approaches accelerate the optimization and simplify the discovery of optimal LNPs. Additionally, we explore novel AI-based strategies for designing superior mRNA delivery systems. The combination of AI and nanomedicine has great translational potential, offering a faster and more efficient pathway for developing efficient, safe, and targeted mRNA delivery systems.

## 2 Challenges in the design of LNPs

Due to the remarkable success of the mRNA vaccines against COVID-19 pandemic, LNPs have emerged as the most promising mRNA delivery vehicles with great clinical translational potential^44^^,^^45^. Beyond utilization in the COVID-19 mRNA vaccines, LNPs also become the leading mRNA delivery platform for a broad range of therapeutic applications, such as cancer immunotherapy, treatment of hereditary disorders, and protection against infectious diseases^1^^,^^2^. As mRNA-based therapies continue to expand, the demand for LNPs has surged, necessitating more sophisticated designs to enhance their efficiency, specificity, and safety. Although the feasibility of the mRNA-LNP delivery systems has been validated, several challenges remain in the design and optimization of LNPs, including screening the chemical structures of lipids, overcoming the liver tropism of LNPs to achieve organ/tissue-specific targeting, and further understanding the complex *in vivo* delivery mechanism^46^, ^47^, ^48^. Traditional experimental methods for optimizing LNPs are often labor-intensive, time-consuming, and require complex data analysis, thereby limiting the development process to a certain extent. With the development of AI and ML, researchers are exploring how to use these advanced computing tools to effectively solve bottlenecks in biomedical development, thereby accelerating the drug development pipeline. To cope with the above limitations, AI has served as an important facilitator for analyzing and processing large amounts of complex data, allowing for more efficient development of LNP-based mRNA delivery systems. With the unique advantages of analyzing the generalized results and predicting the trends, AI provides a promising solution for accelerating the LNP development.

### 2.1 Lipid structural design and optimization of LNPs

Typical LNPs are usually composed of four components: ionizable lipids, phospholipids, cholesterol, and PEG-lipids^20^^,^^49^. Studies have shown that different chemical structures and formulations may affect the trafficking of nanoparticles *in vivo*^50^^,^^51^. Minor alteration in lipid compositions may affect their physicochemical properties, such as particle size, surface charge, and mRNA binding efficiency, all of which can influence mRNA delivery efficiency and organ targeting^52^, ^53^, ^54^. Thus, the major challenges in LNP design include optimizing the molecular structures of ionizable lipids, screening for optimal formulations, and improving functionality to ensure maximum delivery efficiency, organ-specific targeted delivery, and minimal toxicity^55^^,^^56^. However, the precise screening and optimization of these lipid components is a complex process. Traditional experimental approaches to optimize lipid formulations are time-consuming and often fail to rapidly reveal the most effective lipid combinations, given the large number of potential lipid candidates. AI and ML algorithms can be trained on large datasets of lipid formulations and their associated performance metrics to optimize LNP lipid compositions. For instance, deep learning (DL) models can analyze the relationship between lipid chemical structures and LNP characters, such as size, p*K*_a_, zeta potential, mRNA encapsulation efficiency, and transfection efficiency^40^. By utilizing these models, researchers enable the prediction of the most effective lipid combinations for a specific therapeutic target in a shorter time, increasing the efficiency and accuracy of the optimization process.

### 2.2 Realizing organ-specific targeting of mRNA-LNP delivery systems

The mRNA-LNP delivery systems have shown great promise across various disease applications, with the potential for precise targeted therapy through optimized delivery strategies^26^^,^^57^. However, a major issue in LNPs is their liver tropism post systemic administration. This hepatic tropism primarily arises because intravenously administered LNPs bind to apolipoprotein E (ApoE) in the bloodstream, which subsequently interacts with low density lipoprotein (LDL) receptors in the liver, leading to preferential liver accumulation^58^, ^59^, ^60^. While this property is beneficial for liver-targeted therapy, it limits the broader therapeutic potential of LNPs for targeting other organs, such as the lung, spleen, heart, and tumors^51^^,^^61^. Several strategies have been identified to achieve organ-specific targeting, including altering the chemical structures of lipids, changing the surface charges of LNPs, adjusting the compositions of LNPs, and modifying the surfaces of LNPs^46^^,^^50^^,^^62^, ^63^, ^64^. However, since LNPs function in the complex biological environment, predicting how these alterations influence LNP distribution, cellular uptake, protein expression, and targeting remains challenging. Encouragingly, AI offers a powerful solution to predict how these changes in LNPs will affect their biodistribution and targeting by learning from large amounts of experimental data. Computational models can simulate the interactions of LNPs with various tissues or cell types to predict how lipid composition, particle size, and surface charge will affect the cellular uptake and tissue distribution^65^^,^^66^. By incorporating data from biological sources, such as proteomics, transcriptomics, or immune system interactions, AI models can predict how different LNP formulations target specific organs or reduce liver accumulation.

### 2.3 Understanding the mechanism of intracellular delivery of LNPs

Despite the widespread use of LNPs in mRNA delivery, research on the mechanisms of their *in vivo* behavior is still relatively limited. Once administered, LNPs undergo a series of interactions with immune cells, plasma proteins, and various tissues and cells^67^, ^68^, ^69^. Specifically, due to the complex *in vivo* environment, the processes of cellular uptake, endosomal escape and mRNA release after LNP entry are very complicated and not yet fully elucidated. These biological processes are critical in determining the ultimate efficiency of mRNA delivery^70^. Therefore, a deeper understanding of the LNP interactions in the body is essential for optimizing their design to enhance the efficacy and safety. ML provides a way to understand the complicated *in vivo* processes of mRNA-LNP delivery. By analyzing large datasets from high-throughput screening and cell imaging, ML algorithms can identify key factors that affect endosomal escape, cell transport, and immune response^16^. For instance, DL-based image analysis can track the behavior of LNPs in living cells, providing real-time insight into the processes of internalization, endosomal escape, and mRNA release^71^^,^^72^. These advanced tools enable the identification of new mechanisms of action, expanding the application potential of LNPs.

Traditional methods for determining the top-performing ionizable lipids and LNPs rely on extensive screening and trial-and-error experiments^38^^,^^66^. While such screening yields accurate results, it is time-consuming and requires substantial animal use, struggling to analyze the vast complexity of chemical structures and data correlations. These limitations are expected to be addressed with the help of AI. AI, especially ML and DL, is radically changing the optimization process of LNPs by offering tools to systematically analyze and predict the complex relationships between lipid compositions, formulation parameters, and functional outcomes. By integrating AI into LNP optimization, researchers can reduce the dependence on traditional trial-and-error methods, accelerate the discovery of optimal formulations, and significantly improve the accuracy and efficiency of LNP design. DL models, such as deep message-passing neural networks (D-MPNNs), are particularly valuable for screening vast lipid libraries, already assisting in the identification of novel antibiotics and ionizable lipids^65^^,^^73^^,^^74^. AI-driven insights not only improve delivery efficiency, but also enable the development of personalized LNP formulations tailored to specific therapeutic needs, providing a promising approach for advancing mRNA therapeutics.

## 3 AI-guided applications in nanomaterial design

Given the complexity and interdependence of constituents in nanomedicine formulations, addressing above challenges requires more efficient screening methods rather than just relying on traditional trial-and-error approaches. In this context, AI has emerged as a powerful tool that can learn from large datasets, identify potential correlations and predict optimal formulations. By utilizing AI, researchers are beginning to shift the nanomedicine design process from experience-based optimization to a more systematic, data-driven strategy. The following section explores how AI, specifically ML algorithms, can be applied to nanomaterial design and how these approaches can be extended to overcome limitations in current LNP development.

ML, the predominant paradigm in contemporary AI research, is undergoing revolutionary advancement^35^^,^^75^. Its theoretical origins trace back to the threshold-based artificial neuron model proposed by Warren McCulloch and Walter Pitts in 1943^76^. This seminal work not only established the mathematical foundation for neural computation, but also provided a crucial basis for developing subsequent ML frameworks. From the 1950s to the mid-1970s, propelled by breakthroughs in natural language processing, the development of intelligent systems based on ML principles entered its first golden age, attracting extensive academic attention. From the 1990s to the early 21st century, revolutionary developments in ensemble learning theory and kernel methods enabled ML models to demonstrate exceptional generalization capabilities in feature space mapping and high-dimensional data classification tasks^77^^,^^78^. In subsequent technological evolution, feature engineering and model hyperparameter optimization emerged as critical pathways for enhancing algorithmic efficacy^79^, ^80^, ^81^. Currently, ML has become deeply embedded throughout biomedical research, playing indispensable roles in pivotal domains including drug target discovery, drug synergy prediction, and delivery system optimization^82^, ^83^, ^84^.

**Figure 2:**
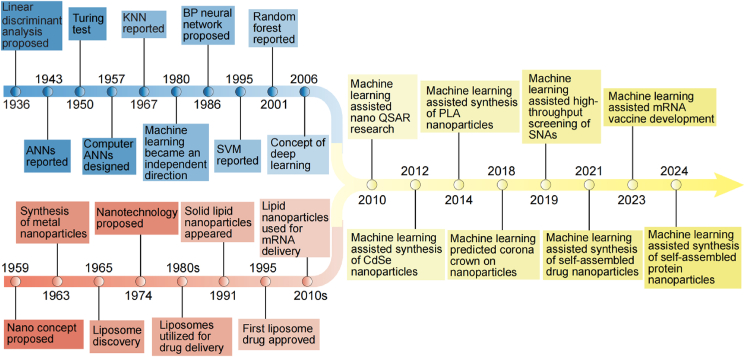
Timeline of key milestones for AI, nanoparticles, and their combination.

The foundations of nanotechnology trace back to 1959, when Nobel laureate physicist Richard Feynman articulated his visionary lecture “There's Plenty of Room at the Bottom” hypothesizing precise atomic-scale manipulation—an intellectual precursor now recognized as nanotechnology's conceptual genesis^85^. The formal term “nanotechnology” subsequently emerged through Norio Taniguchi's systematic framework in his 1974 seminal technical paper^86^. The 1980s witnessed breakthrough translation with liposomal systems emerging as pioneering drug carriers, leading to the 1995 regulatory milestone of Doxil, the first FDA-approved nanopharmaceutical formulation^87^. Entering the 21st century, paradigm-shifting advances in ML have propelled nanomaterials innovation into an accelerated discovery phase. These computational approaches now enable predictive modeling of nanoparticle-protein corona interactions, intelligent optimization of spherical nucleic acid architectures, and precision engineering of LNP-based mRNA vaccines, fundamentally transforming nanosystem design through machine-augmented molecular informatics (Fig. 2)^4^^,^^88^^,^^89^.

### 3.1 The classification of ML algorithms

ML can be categorized into four types, primarily determined by the amount and type of supervision during the training process: supervised learning, unsupervised learning, semi-supervised learning, and reinforcement learning (Table 1)^90^. Within supervised learning, the training data given to the algorithm includes the desired outputs, which are clearly labeled. Supervised learning involves both independent and dependent variables and can be used for classification and regression tasks^91^^,^^92^. Unsupervised learning uses training data that is unlabeled, and the algorithm learns automatically without guidance. In this case, the data only includes independent variables without dependent variables^93^^,^^94^. Semi-supervised learning methods typically rely on certain assumptions about the data. The performance of these algorithms is guaranteed only when these assumptions are met, which is one of the main challenges limiting the widespread application^95^^,^^96^. Reinforcement learning is a learning mechanism that aims to learn optimal behavior by mapping states to actions that maximize the rewards obtained. Such an agent requires constant conduct of experiments in the environment and optimizes the correspondence between state and behavior through feedback provided by the environment. Therefore, repeated experimentation and delayed rewards are two key characteristics of reinforcement learning^96^^,^^97^. Diverse ML algorithms and their applications are summarized in Tables 1^98^, ^99^, ^100^, ^101^, ^102^, ^103^, ^104^, ^105^, ^106^, ^107^, ^108^, ^109^, ^110^, ^111^, ^112^, ^113^, ^114^.

**Table 1:** Summary of the ML algorithms and their applications for nanomedicine.

Category	Method	Advantage	Disadvantage	Application	Ref.
Supervised learning	• *K*-Nearest Neighbors (KNN)	• Make no assumptions about the data • Low cost in the learning process	• Dependent on selection of *K* value	• Optimize the preparation conditions of mRNA-LNP vaccines • Advance the prediction of the impact of global AgNPs on soil enzymes	98, 99
	• Support vector machine (SVM)	• Proficient in solving complex learning tasks • No assumptions were made regarding the predictor variables	• Expensive computational costs • Can only handle continuous predictor variables	• Demonstrate the concentration determination of plasmonic nanoparticle mixtures • Highly accurate classification of different crystals	100, 101
	• Random forest (RF)	• Unlikely to overfit • High classification accuracy • Capable of handling missing values	• Slow running speed • Not easy to explain	• Accurately predict the pulmonary immune responses and lung burden of NPs • Improve the efficiency of placental uptake of LNPs	102, 103
	• Naive Bayes	• Can handle continuous and categorical predictor variables • Low training expenses • No hyperparameters need to be adjusted	• Continuous predictor variables need to have a normal distribution • Predictive variables need to be independent of each other	• Predict the potential environmental risks of nanoparticle exposure	104
	• Decision tree (DT)	• Easy to explain • Can handle categorical and continuous predictor variables	• Easy to overfit	• Simulate the cytotoxicity of nanoparticles • Predict the correlation between nanoparticle characterization results and cytotoxicity	105, 106
Unsupervised learning	Clustering				
	• *K*-means	• Scalable and efficient • Local optima can already meet the needs of clustering	• Initial *K* value needs to be determined in advance • *K*-means can only converge to local optima	• Identify all regions with distinct spectral features of nanoparticles • Predict the adsorption free energy of biomolecules on specific nanomaterials	107, 108
	• Expectation maximization algorithm	• Good performance for hybrid models • Not necessary to make assumptions about observational data	• Sensitive to initial values • Difficult to handle missing data • Easy to get stuck in local optimal solutions	• Retrieval of size distributions from the small-angle scattering patterns obtained from dense nanoparticle samples • Shelf-life prediction of nano-sol	109, 110
	Dimensionality reduction				
	• Principal component analysis (PCA)	• Low computational cost • Completely parameter free	• Cannot intervene in the processing process through parameter tuning • Eigenvalue decomposition has some limitations	• Determine the influence of various factors on the preparation of modified nanocomposites	111
	• Kernel PCA	• Overcome the approximate linear structure of data • Enhanced flexibility and adjustability	• High computational complexity • Unable to explain the mapping result	–	
	Association rule learning				
	• Apriori	• Strong interpretability • Strong scalability	• Data sparsity problem • Unable to handle continuous data	–	
	• Eclat	• Easy to parallelize • Space efficiency	• Lack of flexibility • Unable to handle missing values	–	
Semi-supervised learning	• Self-training	• Not dependent on model architecture • Not dependent on the dataset	• Restricted by the distribution and quantity of labeled samples • Difficult to find high confidence unlabeled samples	–	
	• Semi-supervised rotationally invariant variational autoencoder	• Rotational invariance • Compact latent space representation	• Complex implementation • Limited scalability • Potential for latent space discontinuity	• Classify gold nanoparticles with different properties	112
Reinforced learning	• Monte Carlo tree search	• No need for extensive domain knowledge • Suitable for large state spaces	• Strong dependency on search iterations • High computational resource consumption	• Design complex protein nanomaterials	113
	• Machine learning reinforced genetic algorithm	• High adaptability • Improves search efficiency • Suitable for large scale and complex problems	• High computational complexity • Complex parameter tuning • High implementation difficulty	• Targeted search for selective cytotoxic inorganic NPs	114

LNPs, lipid nanoparticles; NPs, nanoparticles; AgNPs, Ag nanoparticles; ‒, not applicable.

### 3.2 The application of ML algorithms

#### 3.2.1 Supervised learning

Random Forest (RF) is a supervised learning algorithm first introduced by Tin Kam Ho in 1995^115^ and then developed by Leo Breiman^116^, which is suitable for both classification and regression problems. RF is an ensemble learning algorithm consisting of multiple decision trees. One of its key features is its ability to reduce overfitting caused by individual decision trees, thereby improving model performance. The basic principle of RF can be summarized in three steps: random sampling, random feature selection, and majority voting^117^^,^^118^.

Due to its superior performance, RF has been widely applied in predicting the delivery efficiency by LNPs. Compared to traditional lipid formulations, LNPs show significant improvements in endosomal escape and encapsulation rates. Additionally, LNPs with specific compositions can cross the placental barrier, transporting immunoglobulin G (IgG) *via* the FcRn receptor to the fetus for immunotherapy^119^. The transfection efficiency of nucleic acids is a key factor in treatment. By inputting a total of 18 features, such as particle size and zeta potential, from 41 LNP formulations and 48 different transport experiments into various models—Extreme Gradient Boosting (XGB), Decision Tree (DT), RF, Support Vector Regression (SVR), Light Gradient Boosting Machine (LightGBM), and Lasso with Least Absolute Shrinkage and Selection Operator (Lasso)—the results demonstrated that the RF model provided the most accurate predictions for nucleic acid delivery efficiency^103^. SHapley additive explanation (SHAP) analysis was used to evaluate feature importance, successfully synthesizing LNPs with low, medium, and high transfection efficiency, thereby offering some explanation for the RF model^103^. Since the coronavirus pandemic, mRNA vaccines have revolutionized the vaccinology field and LNPs have been widely used for mRNA delivery. Bae et al.^38^ used 314 features from 213 LNPs (*e.g.*, the ratio of amine groups of ionizable lipids to phosphate groups of the encapsulating unit, the mass and molar ratio of ionizable lipids, cholesterol, etc.) to train an RF regression model to predict mRNA expression levels after intradermal injection in mice. During the 10-fold cross-validation of the RF regression model, the model achieved optimal performance using 15 features, resulting in a coefficient of determination (*R*^2^) of 0.708 and a Pearson correlation coefficient (PCC) of 0.845, indicating successful prediction of mRNA expression efficiency after intradermal injection. Feature importance analysis identified phenol as the key substructure influencing mRNA encapsulation and expression.

#### 3.2.2 Unsupervised learning and other types of machine learning

Principal component analysis (PCA) is one of the simplest and most fundamental unsupervised learning methods. Its background and development can be traced back to the early 20th century, and it remains a cornerstone in the field of multivariate statistical analysis. PCA was first proposed by Karl Pearson in 1901^120^ and was later further developed by Harold Hotelling in 1933^121^. High-dimensional data often suffer from the “curse of dimensionality”. PCA addresses this by extracting the main features, identifying a lower-dimensional affine subspace and projecting all data points onto it to achieve dimensionality reduction^122^.

Romanò et al.^123^ collected voltammograms of LNPs with three different lipid compositions, standardized the data, and used PCA analysis to extract the main features of the voltammograms (PC1 and PC2). Linear discriminant analysis (LDA) is used for further classification, and different formulations of lipid nanocarriers are distinguished along the PC1 and PC2 directions. Cross validation showed that the method had a prediction accuracy of 90% for three different test formulations, and the first and second principal components explained 98.9% of the data together. This method not only confirms the classification potential of electrochemical signals combined with PCA and LDA, but also provides a low-cost and portable solution for rapid quality control of liposome formulations, significantly improving the automation level of drug carrier development and clinical diagnosis^123^. In the development of nanostructured lipid carriers, lipid solid dispersions, and self-emulsifying drug delivery systems, solid or semi-solid lipid excipients are typically mixed with liquid solvents or lipids to form stable formulations. Even when the excipients visually appear miscible once melting, they may exhibit microscopic heterogeneity, resulting in instability over time. For instance, cetyl palmitate + Transcutol© (heterogeneous) and polyethylene glycol-6000 (PEG-6000) + Tween 80© (homogeneous) exhibited significantly different degrees of microscopic miscibility^124^. These systems were prepared in varying proportions for Raman spectroscopy analysis, and the resulting data underwent PCA. By observing the changes in the PC1 and PC2 directions of different systems, it is possible to distinguish the degree of uniformity of mixing of different compounds in the system.

Due to the more stringent usage conditions of semi supervised learning and reinforcement learning in the LNP field, their applications are currently limited. The critical challenge in developing nanopore biomarker analysis lies in directly identifying and quantifying target biomarkers from biological fluids containing molecular mixtures. Greive et al.^125^ successfully classified peptide conformational variants at the single-molecule level with a semi supervised classification algorithm, which other ML algorithms could not accurately classify. The combination of reinforcement learning and genetic algorithm can improve the accuracy of the algorithm. For instance, using ML enhanced genetic algorithm, selective cytotoxic inorganic nanoparticles can be discovered by screening 14,900 candidate substances, and the accuracy of the model is high (Q1, 0.80; RMSE, 13.6)^114^.

### 3.3 Model recommendation

When studying drug delivery with LNPs, researchers often encounter various scenarios, such as optimizing and predicting drug delivery efficiency, controlling drug release rates, evaluating targeting capabilities, and optimizing LNP formulations^20^^,^^29^^,^^126^. Selecting the appropriate ML model for each scenario can significantly improve the optimization efficiency and effectiveness of LNP delivery systems. We summarize some recommendations for choosing different ML models based on specific scenarios.

When predicting the release rate of a drug from different LNP formulations, certain characterization data are typically available, such as lipid composition, particle size, zeta-potential, and pH. The target variable is usually the concentration or release rate of the drug at a specific time. In this case, ML models tend to favor RF and Gradient Boosting Regression models (*e.g.*, XGBoost and LightGBM). RF can handle complex nonlinear models and is highly sensitive to interactions between features. It is well-suited for processing high-dimensional data and can provide feature importance scores, helping to identify which factors show the most significant impact on the release rate. Gradient Boosting Regression models can continuously optimize the model during the iterative process, gradually reducing errors and achieving higher prediction accuracy, especially when dealing with complex nonlinear relationships^127^^,^^128^.

A key factor of successful LNP delivery is their internalization by targeted cells. Gandek et al.^129^ indicated that LNPs primarily internalized through classical endocytosis mechanisms. When predicting whether different LNPs can be taken up by certain cells, the Support Vector Machine (SVM) model is often a suitable choice. SVM can learn the causal relationship between the properties of different LNPs and their cellular uptake. SVM is capable of handling high-dimensional data and can find better decision boundaries for linearly inseparable data through kernel functions^130^. In the context of LNP cellular uptake, where cellular responses may not be linear, SVM is particularly effective in addressing such issues.

The targeting ability of LNPs represents another important issue of concern. Predicting whether LNPs can target specific cells or organs based on their characterization results is a multiclass classification task, for which RF is recommended^38^. RF is not only suitable for regression tasks but is also highly effective for classification tasks. However, the XGBoost model often shows higher accuracy in handling multiclass tasks with class imbalance. The Multi-Layer Perceptron (MLP) is a basic neural network framework and is also applicable to multiclass problems. By increasing the number of layers, MLP can capture complex nonlinear relationships^131^^,^^132^. In case of large and complex datasets, MLP should be prioritized as the model of choice.

Some reinforcement learning models, such as Deep Q Networks (DQN) and Q-learning, are less used in LNP research. Optimizing the LNP formulations to maximize drug delivery efficiency and targeting is a dynamic optimization problem that may involve multiple experimental feedbacks. Each parameter in the formulation may potentially influence the final drug delivery outcome. Reinforcement learning is well-suited for decision optimization in feedback environments^133^, which can continuously adjust the LNP formulations based on experimental results to achieve the remarkable delivery performance.

The choice of different models and dataset sizes can result in different costs and time requirements. In this context, multiple models can be tested for the task, allowing the best model for subsequent analysis based on the result accuracy and the model's performance.

## 4 AI-guided key techniques and methods in LNP design

### 4.1 AI-guided optimization of LNP compositions and formulations

The LNP properties, such as particle size, polydispersity (PDI), zeta potential, and p*K*_a_, play critical roles in determining their delivery capabilities^52^. The composition and formulation of LNPs represent the critical factors that govern these characteristics. To apply LNPs in mRNA therapies, extensive screening of nanoparticle structure, composition, and formulation is typically required. Given the complexity and high number of variables involved, conventional experimental methods are both time-consuming and resource-intensive. Here, AI offers a powerful tool to analyze and uncover hidden correlations within large and complex datasets. Leveraging AI's capacity to process massive amounts of data and recognize patterns, researchers can predict the most promising lipid compositions and formulations for optimal mRNA delivery. This not only accelerates the development of LNPs, but also significantly improves the efficiency and accuracy of the design process.

#### 4.1.1 Optimization of the LNP components

**Figure 3:**
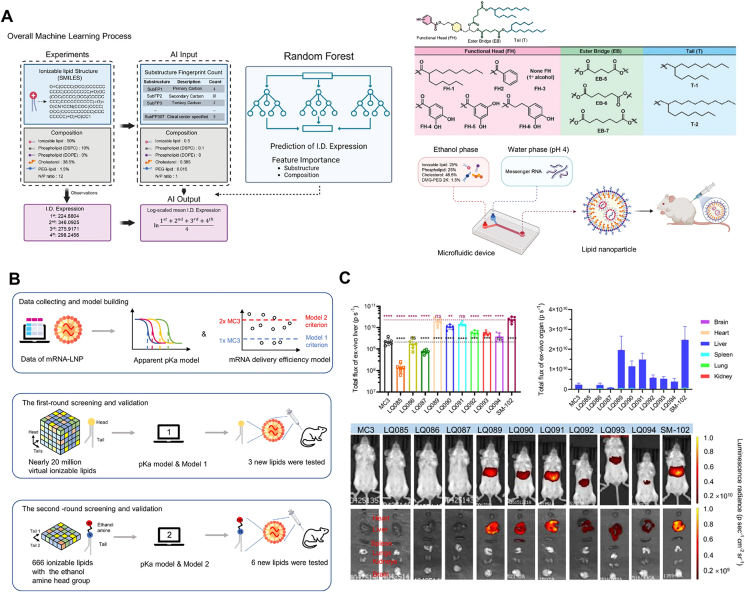
AI-guided key processes in LNP design. (A) A schematic diagram of predicting the mRNA delivery efficiency and assessing the importance of characteristics in ionizable lipid substructures and LNP compositions through ML. Reprinted with the permission from Ref. 38. Copyright © 2024 Wiley. (B) Schematic diagram of rational design of ionizable lipids driven by AI. The acquired data were used to construct and train a model to analyze the potential association between the apparent p*K*_a_ of LNPs and their mRNA delivery efficiency. (C) Organ expression of luciferase mRNA after intravenous injection of new LNPs designed *via* AI guidance. Reprinted with the permission from Ref. 66. Copyright © 2024 Springer Nature.

The LNP design for mRNA delivery requires careful selection and optimization of various components, including ionizable lipids, phospholipids, cholesterol, and PEG-lipids^20^^,^^28^. Ionizable lipids, the core component of LNPs, remain neutral under physiological conditions but become protonated and positively charged in acidic endosomes, thereby reducing the lipid toxicity^28^^,^^32^^,^^134^. Optimizing ionizable lipids typically involves altering the tail lengths, the head-group chemical structures, and the linker groups^135^. These adjustments are essential for enhancing the mRNA encapsulation efficiency and endosomal escape of nanoparticles. Li et al.^39^ proposed an approach combining ML and combinatorial chemistry to accelerate the discovery of novel ionizable lipids for optimizing mRNA. The authors used the binary dataset to evaluate the performance of different ML algorithms, including RF, gradient boosting and logistic regression. The results demonstrated that XGBoost algorithm presented superior predictive performance, with the highest PR curve of 0.987 and ROC curve of 0.983. High-throughput screening (HTS) and ML prediction resulted in the discovery of novel ionizable lipid 119-23 with excellent performance from a large chemical library, whose mRNA delivery efficiency outperformed existing commercially available lipids (*e.g.*, DLin-MC3-DMA (MC3) and SM-102). Bae et al.^38^ utilized a branching criterion under the RF model to assess the importance of individual features on mRNA expression. The FP169 (phenol) was identified as the optimal functional head group in ionizable lipid libraries, with the highest importance score of 0.297. The hydroxyl group in phenol enhanced the interaction with mRNA, thereby improving both the efficiency and stability of mRNA-LNPs (Fig. 3A)^38^.

Phospholipids are typically used as the helper lipids in LNPs and serve to maintain the stability of nanoparticles^27^^,^^136^. Recent research has focused on modifying phospholipids to improve their interaction with ionizable lipids and enhance the overall performance of LNPs^137^. Cholesterol is another essential component of LNPs and enhances the stability of LNPs. It improves the structural integrity of the lipid bilayers, increasing the overall particle stability and protecting encapsulated mRNA from degradation^20^^,^^52^^,^^138^. Researchers have explored the possibility of modifying the cholesterol structure or substituting it with other sterols for enhanced safety and delivery efficacy^139^, ^140^, ^141^. Although PEG-lipids account for the lowest molar percentage of LNPs, they exert influences on many key properties of LNPs, including particle size and dispersion, nanoparticle stability, and their biodistribution and blood circulation time^52^^,^^142^. Positioned on LNP surfaces, PEG-lipids can reduce the protein coronal adsorption and recognition by the immune system, prolonging the LNP circulation time in bloodstream and modulating the particle biodistribution *in vivo*^28^^,^^59^. Mui et al.^142^ discovered that the chain length and concentration of PEG-lipids significantly affected the *in vivo* journey and delivery efficiency of LNPs, with longer carbon chains of PEG-lipids extending blood retention. Since adjustments between components in LNPs are delicate and impactful, researchers are investigating various properties between fine-tuned components to optimize mRNA delivery. While current AI-guided design of LNPs primarily focuses on optimizing ionizable lipid libraries and formulations, the potential extends far beyond these applications. In the future, it is expected that AI will also be applied to the screening of phospholipids, sterols, and PEG-lipids, providing a more comprehensive approach to optimize LNPs.

AI is a potent tool for accelerating LNP design and screening process, allowing rapid evaluation of lipid chemical libraries and identification of optimal formulations^39^^,^^143^. ML models, especially those based on supervised learning, have been used to predict the efficacy of different lipid combinations based on historical data and experimental results^35^^,^^144^. For instance, algorithms including RF, SVM, and neural networks have been utilized to associate the lipid chemical structures to their mRNA encapsulation efficiency, cellular uptake, and transfection efficiency. This dynamic approach allows for the continuous improvement of LNP design by autonomously identifying and promoting the most effective formulations.

The integration of AI into LNP research not only accelerates the discovery of novel lipid structures, but also promotes the design of highly efficient, targeted delivery systems for mRNA therapeutics. By utilizing data-driven insights and predictive modeling, AI-driven methodologies demonstrate potential to revolutionize nanomedicine and enable the rapid translation of advanced lipid-based drug delivery systems from the laboratory to the clinic.

#### 4.1.2 Analysis and prediction of LNP physicochemical properties by ML

As an important tool for analyzing and predicting the physicochemical properties of LNPs, ML can help to provide a deeper understanding of how lipid compositions affect their structural and functional properties^66^. Trained by inputting a large amount of data from experimental and computational results, ML can reveal the complex correlations between lipid compositions and key physicochemical parameters (*e.g.*, particle size, surface charge, p*K*_a_, mRNA encapsulation efficiency, stability, and biodegradability), and predict physicochemical parameters that are more conducive to mRNA delivery.

The physicochemical properties of LNPs are governed by a variety of factors, including lipid structures, component molar ratios, and formulation conditions. Traditional experimental methods for characterizing and analyzing these properties are often complicated and incomprehensive. ML algorithms provide a way to analyze high-dimensional datasets and identify key predictors of LNP behavior with high accuracy. For instance, particle size and PDI are key factors influencing LNP stability and biodistribution. ML models trained on formulation parameters and experimental measurements can accurately predict these properties, facilitating rapid optimization of nanoparticles. Maharjan et al.^127^ employed ML algorithms, including XGBoost, Bayesian Optimization, and Self-Validated Ensemble Model (SVEM), to improve productivity and optimize LNP formulations. They utilized XGBoost to comprehend the significance of various LNP parameters and predict the particle size, PDI, zeta potential, p*K*_a_, heat trend cycle, and mRNA encapsulation rate of LNPs. The results showed that the XGBoost model possessed excellent predictive capability, with predictive response higher than 94%. Similarly, p*K*_a_ prediction is useful for assessing carrier stability and *in vivo* organ targeting, thereby guiding the screening of stable, low-toxicity, and targeted delivery systems^47^. In addition, AI models can also speed up the screening process of ionizable lipids by effectively predicting the p*K*_a_ and mRNA delivery efficacy. Wang et al.^66^ used the LightGBM algorithm to construct a p*K*_a_-based model and successfully screened the ionizable lipid LQ089 with higher mRNA delivery efficiency than the commercial lipid DLin-MC3-DMA and comparable efficacy to SM-102. The LightGBM model showed superior performance over the RF algorithm in several parameters, with its Recall of 0.86 and its accuracy (ACC) of 0.90. Their results demonstrated that effective mRNA delivery was often associated with LNP p*K*_a_ in the range of around 6.0 to 7.0 (Fig. 3B and C)^66^. By analyzing experimental degradation data, supervised learning techniques can predict changes in size, surface charge, mRNA encapsulation efficiency, and delivery efficiency over time, providing insights into formulation stability and fueling the LNP development.

### 4.2 AI-guided understanding of LNP *in vivo* targeting

#### 4.2.1 Targeting guided by nano-bio interactions

**Figure 4:**
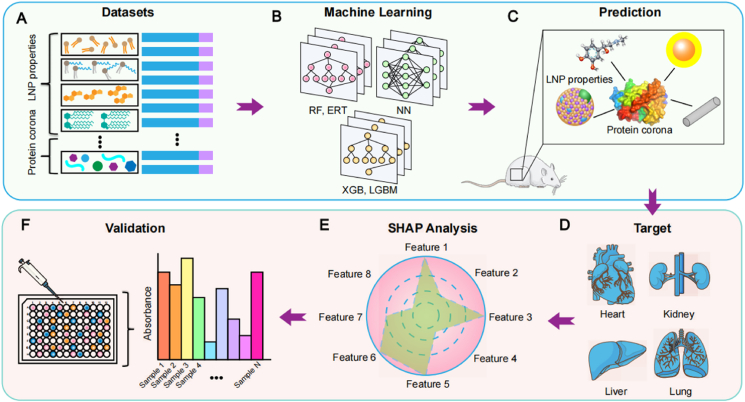
The process of AI-guided understanding of *in vivo* targeting of LNPs. (A) Organization of the LNP characteristic and protein corona datasets. (B) Training of different machine learning models using data. (C) Prediction of LNP properties and protein corona. (D) Organ targeting prediction of LNPs. (E) Identification of key features by SHAP analysis. (F) Experimental verification of predicted LNPs.

Once entering the body, LNPs interact with cells, tissues, and other components. These interactions determine the fate of LNPs in the body, deciding whether they accumulate in a target organ, tissue, or cell and exert therapeutic effects at specific sites (Fig. 4)^16^^,^^145^. Therefore, understanding the factors that influence the LNP targeting is essential for precision therapy. ML algorithms offer unique advantages in data clustering, classification, and prediction. By performing high-throughput analysis on existing data, these algorithms can not only accurately predict the targeting efficiency of LNPs, but also guide the synthesis of new targeted LNPs.

The targeting ability of LNPs largely depends on their properties, such as size and composition. Smaller nanoparticles show a stronger capacity to penetrate the endothelial barrier and enable passive targeting to the tumor tissues *via* the enhanced permeability and retention (EPR) effect^146^. Larger nanoparticles tend to be captured by the liver-spleen mononuclear phagocyte system (MPS), making them suitable for liver-targeted delivery, such as in siRNA or mRNA-based liver therapies^147^. The core composition of LNPs includes four types of lipids, and the ratio and type of each lipid directly affect their targeting efficiency^52^. For instance, cholesterol plays a critical role in modulating membrane fluidity, enhancing the stability and mRNA encapsulation efficiency. A higher proportion of cholesterol aids in LNP uptake by liver cells, while a lower cholesterol content may reduce hepatic accumulation^148^^,^^149^.

**Figure 5:**
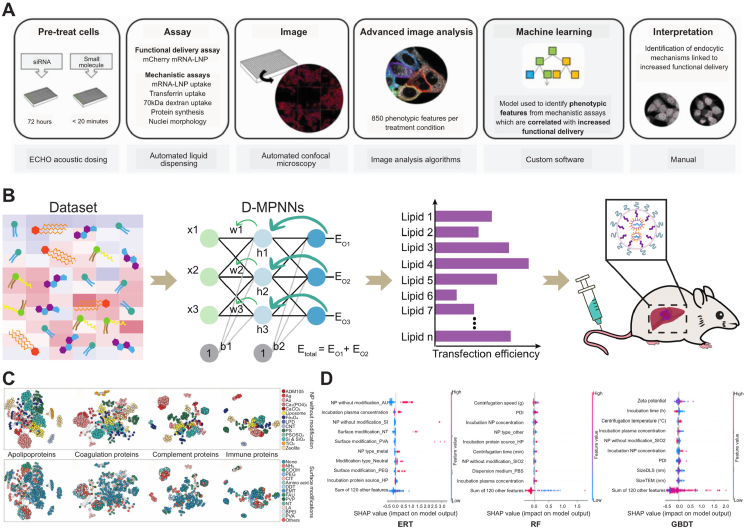
AI-guided prediction of LNP targeting. (A) A workflow that combined advanced image analysis with ML. The workflow included the following steps: firstly, cells were treated with siRNA or small molecules to obtain advanced images of the cells, which were then input into a ML model. Finally, ML methods were used to predict the mRNA delivery efficiency of LNPs. Reprinted with the permission from Ref. 150. Copyright © 2023 Wiley. (B) Prediction workflow between lipid chemical structure and delivery efficacy. The workflow included the following steps: firstly, database data was organized and the D-MPNN model was trained. Then, the model was used to predict the relationship between the structure and delivery efficacy of external data. Finally, the delivery effectiveness of selected lipids was verified. (C) The heterogeneity of functional corona compositions across the models was visualized through the similarity network. Reprinted with the permission from Ref. 89. Copyright © 2020 National Academy of Sciences USA. (D) An analysis of the individual feature contributions in ERT, RF, and GBDT models. Reprinted with the permission from Ref. 153. Copyright © 2024 the American Association for the Advancement of Science.

Hunter et al.^150^ pioneered an integrated approach combining advanced image analytics with ML to enhance the targeted delivery of LNPs (Fig. 5A)^150^. Utilizing high-content image analysis, they systematically extracted 850 multidimensional phenotypic features from siRNA- or compound-treated cell populations. These parameters encompassed cellular density, nuclear-to-cytoplasmic ratio, and spatially resolved fluorescence intensity distributions of subcellular markers. Three ML architectures—RF, Gradient Boosted Decision Trees (GBDT), and KNN—were employed to analyze the standardized feature dataset for identifying biomarkers predictive of optimized LNP targeting. The final predictive models demonstrated robust performance with 74%–81% overall accuracy and 66%–75% class-specific accuracy. Feature importance analysis revealed 10 high-impact determinants, including nuclear envelope curvature dynamics and topological reorganization of perinuclear 70 kDa dextran-positive vesicles.

By systematically integrating 20 multi-source datasets encompassing over 9000 LNP activity measurements, Witten et al.^65^ developed a D-MPNN model to establish predictive relationships between lipid chemical structures and delivery efficacy (Fig. 5B). This framework demonstrated exceptional extrapolation capability to structurally distinct compounds beyond the training set. Through virtual screening of 1.6 million candidate lipids, two novel ionizable lipids (FO-32 and FO-35) were identified. Experimental validation revealed that FO-32 achieved benchmark performance in murine pulmonary nebulization delivery, while FO-35, featuring an innovative terpene-derived tail architecture, accomplished unprecedented transfection efficiency across the full pulmonary epithelium of ferrets—a critical preclinical model for respiratory therapeutics. Notably, these optimized lipids contained chemically counterintuitive functional group combinations (*e.g.*, alkyne motifs with branched carboxylic acid tails) that conventional rational design approaches would unlikely predict, thereby demonstrating DL's unique capacity to transcend traditional chemical space exploration limitations. This study establishes that ML not only enhances the screening efficiency for existing lipid libraries but also enables *de novo* design of four-component reaction systems, creating a novel paradigm for developing targeted gene delivery vehicles against respiratory diseases.

#### 4.2.2 Targeting guided by protein corona

The protein corona, a dynamic biomolecular adsorption layer formed on nanoparticle surfaces upon entering biological microenvironments, governs key pharmacological determinants ranging from systemic circulation half-life and immune evasion capacity to tissue-specific biodistribution^67^^,^^151^. Conventional corona characterization relying on mass spectrometry and *in vitro* simulation faces critical limitations in predictive accuracy and design capability due to experimental throughput constraints, interspecies variability, and dynamic evolution complexity. Recent advances in AI have revolutionized this paradigm through systematic integration of nanoparticle physicochemical descriptors (size, zeta potential, hydrophobicity), protein interactome data, and multi-omics profiles^127^^,^^152^. ML architectures now enable deconvolution of the tripartite “nanoparticle–corona–biological barrier” interaction network. Notably, graph neural network-based frameworks have achieved probabilistic modeling of corona composition under specified physiological conditions, while reinforcement learning algorithms demonstrate potential for reverse-engineering surface modification strategies to promote preferential adsorption of targeting proteins (*e.g.*, apolipoproteins, transferrin). Emerging AI-driven multiscale modeling transcends the classical “passive corona formation” dogma by simulating dynamic corona evolution coupled with membrane receptor cooperativity, establishing mechanistic foundations for designing adaptive targeting delivery systems with microenvironment-responsive intelligence.

In a prior study, a total of 652 pieces of data associated with protein corona on diverse nanoparticles (NPs) were extracted and analyzed (Fig. 5C)^89^. Eight qualitative characters (*e.g.*, NP type, NP shape, and unmodified NPs) and 13 quantitative factors (*e.g.*, pH of dispersion medium, zeta potential, PDI, and plasma concentration for incubation) were incorporated into the analysis. Using a RF model combined with 10-fold cross-validation, this study comprehensively examined the relationships between the physicochemical characters and biological functions of protein coronas on the NP surface. The results indicated that unmodified NPs and surface modifications were the two most critical characters that determined the formation of protein corona. The ML model achieved high predictive accuracy (*R*^2^ values mostly above 0.75) in estimating the relative protein abundance (RPA) of key functional proteins in the corona, such as immune proteins, complement proteins, and apolipoproteins^89^. Furthermore, the model successfully predicted cell recognition processes mediated by the protein corona, including macrophage uptake and cytokine release. Supported by experimental validation, this study unveiled the pivotal role of functional epitopes within the protein corona in modulating the biological recognition and targeting efficacy of NPs. This novel approach addresses the limitations of traditional models in predicting protein corona formation mechanisms under complex multidimensional conditions, significantly reduces experimental workload, and provides an efficient tool for elucidating nanoparticle–protein interface interactions as well as designing targeted nanomedicines.

In addition, a multitasking ML framework was employed to predict the RPA of proteins within the protein corona (Fig. 5D)^153^. This study utilized binary classification models, such as extremely randomized trees (ERT) and RF, to determine whether a protein adsorbs onto nanoparticles, followed by regression models (ERT, RF, and GBDT) to predict the specific RPA values. Through SHAP interpretability analysis, it was revealed that features such as “unmodified nanoparticle types” (*e.g.*, Au and SiO_2_), “incubation protein source” (*e.g.*, human serum and HS), and “centrifugation temperature” significantly influenced the prediction outcomes^153^. This research provided a data-driven strategy for designing targeted nanoparticles: by harnessing ML to identify key features. Parameters, such as nanoparticle surface modifications and incubation conditions, can be optimized to selectively modulate the protein corona composition, thereby enhancing the targeting efficiency of nanocarriers to specific organs or cells. This work not only validated the advantages of ML in protein corona prediction (*e.g.*, achieving an AUROC of 0.97 in classification tasks and an *R*^2^ of 0.53 for regression tasks using the ERT model) but also offered a novel approach for “active protein corona design to enable precision targeting” in nanomedicine.

### 4.3 AI-guided prediction of LNP trafficking *in vivo*

The *in vivo* transport of LNPs is an intricate process influenced by numerous factors, including the *in vivo* circulatory environment, tissue biodistribution, cellular uptake, and intracellular translocation. An in-depth understanding of these interactions is imperative for optimizing mRNA-LNP drug delivery systems. This process typically requires characterizations by multiple tools, such as imaging, multi-omics studies, and pharmacokinetic analysis. AI-driven methodologies, encompassing ML and DL algorithms, offer potent tools for predicting and optimizing these processes, consequently facilitating more efficient and targeted LNP design.

#### 4.3.1 AI-guided prediction of the biodistribution and mRNA expression by LNPs *in vivo*

The mRNA-LNP biodistribution and expression are critical determinants of therapeutic efficacy, presenting how effectively the mRNA translates into functional proteins within target tissues. Post administration, LNPs circulate systemically, interact with plasma proteins, immune cells and the endothelial barrier, and accumulate in specific organs. The biodistribution of LNPs is affected by factors such as particle size, lipid composition, PEGylation and surface charge^47^^,^^50^^,^^154^. The traditional method of evaluating the *in vivo* distribution of mRNA-LNPs is to inject fluorescently labelled mRNA and trace it using an *in vivo* imaging system (IVIS). This measurement is usually time-consuming with limited scope. However, AI-based models can integrate large-scale datasets from preclinical and clinical studies to establish a more accurate predictive framework for the distribution of mRNA-LNPs.

**Figure 6:**
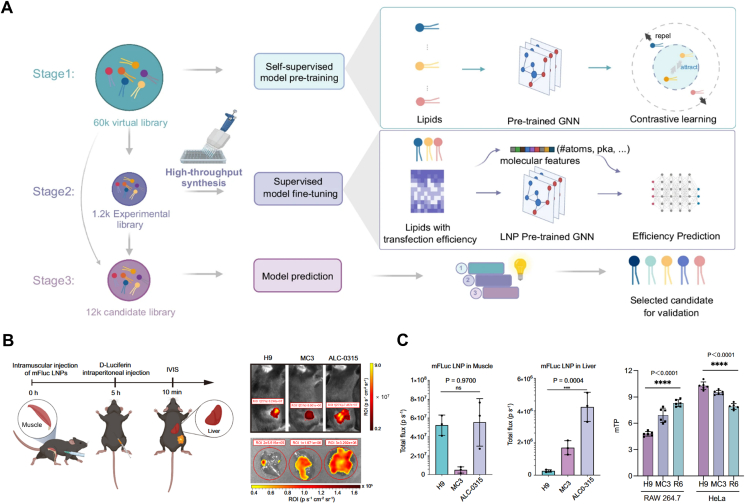
AI-guided prediction of the *in vivo* process of LNPs. (A) Illustration of the workflow designed by the AGILE platform. The workflow of the AGILE platform included the following steps: firstly, the model was pre-trained through self-supervision with a virtual library; then, the model was adjusted through supervised learning with laboratory data; finally, the model is applied to analyze and predict the candidate lipid library. (B) IVIS imaging exhibited the delivery efficiency of intramuscularly injected mFluc LNPs *in vivo* (0.5 mg/kg). (C) Comparison of H9, MC3 and ALC-0315 LNP-mediated mRNA delivery in the muscle and liver *via* intramuscular injection. The mRNA delivery in RAW 264.7 and HeLa cells was also shown. Reprinted with the permission from Ref. 40. Copyright © 2024 Springer Nature.

The ultimate goal of LNP-based mRNA delivery is efficient protein expression in targeted cells. After reaching the targeted tissue, LNPs must be taken up by the cells, escaped from the endosomes, and release the mRNA cargo in cytoplasm for translation. AI-driven models help to predict and optimize these processes. Graph neural networks (GNNs) have strong structure-awareness capabilities, which and can efficiently process complex molecular structures and learn the relationships between molecular structures^40^^,^^155^. Supervised learning algorithms trained on experimental gene expression datasets can predict how different LNP structures and formulations affect mRNA translation efficiency in different tissues^40^. These models take into account factors such as lipid composition, chemical structures, nanoparticle size, and cell tropism to estimate expression levels and duration. For instance, Xu et al.^40^ proposed the AI-Guided Ionizable Lipid Engineering (AGILE) platform, which combined DL and combinatorial chemistry to greatly expedite the development process of LNPs (Fig. 6A). In this study, 60,000 lipids were trained by self-supervised learning and combined with high-throughput experimental data to fine-tune the model, which was able to accurately predict the mRNA transfection efficiency. Finally, the lipid H9 for efficient muscle delivery and R6 for high macrophage transfection were successfully screened (Fig. 6B and C)^40^. In addition, AI-driven molecular dynamics (MD) simulations can help to identify lipid structures that enhance mRNA stability and improve mRNA expression efficiency^152^^,^^156^. A previous study by Gao et al.^157^ simulated the LNP assembling in neutral buffer by MD, and the results revealed that the encapsulation efficiency increased along with the increase of N/P ratio, with nucleic acid completely encapsulated at N/P ratio of 6.

In addition, AI can help to design LNPs that optimize organ-specific expression by analyzing biodistribution data and tissue-specific transfection rates. For example, transformer-based models can predict how changes in lipid composition affect mRNA uptake and translation in hepatocytes *versus* muscle^38^^,^^40^ or lung tissue^65^. These insights aid in developing precise nanomedicine approaches to ensure that LNP formulations are tailored for specific therapeutic applications, such as vaccines, and tissue-specific medicines.

#### 4.3.2 AI-guided prediction of the immune response and inflammation

The immunogenicity of LNPs presents a double-edged sword. On the one hand, LNPs can enhance the vaccine efficacy by inducing helper T cells and promoting humoral response, acting as vaccine adjuvants^158^^,^^159^. On the other hand, they may trigger inflammation and lead to side effects (*e.g.*, inflammation at the injection site or a systemic inflammatory response)^160^. LNP-induced immune responses and inflammation affect their safety and efficacy^161^. While a certain degree of immune activation is desirable in vaccine applications, excessive or unintended immune responses can result in adverse reactions, rapid clearance, and reduced therapeutic efficacy^162^. With its powerful data extraction and data analysis capabilities, AI has been applied in the field of immunotherapy, enabling the analysis of parameters affecting the immune response^38^^,^^163^^,^^164^. Bae et al.^38^ delved into the effect of molecular substructure on the immune response of the mRNA-LNP delivery system by AI predictions. They discovered that the number of phenolic hydroxyl groups was intimately associated to the total IgG level and the expression level of inflammatory factors. AI-driven methods can analyze and predict the immunogenicity of the vectors through the results of prior extensive experiments, enabling rational design of LNPs to strike a balance between immune activation and biocompatibility.

Upon administration, LNPs interact with diverse constituents of the immune system, including macrophages, dendritic cells, and complement proteins. These interactions can trigger inflammatory cytokine release, pattern recognition receptor (PRR) activation, and adaptive immune responses^165^. LNP-based AI models trained on immunological datasets can predict how different LNP formulations affect these immune pathways, thus facilitating the design of nanoparticles with optimized immunogenicity profiles. Wang et al.^152^ utilized the LightGBM ML algorithm to predict the mRNA vaccine-induced IgG titer, an important measure of vaccine immunization efficacy, and the model performed well for accelerating the development of mRNA vaccines. The results showed that higher N/P ratio (*e.g.*, 6:1) was usually correlated with higher mRNA delivery efficiency and enhanced antigen expression. In addition, the inflammation induced by LNPs is a key factor for evaluating their safety and efficacy. Inflammatory responses can be predicted by AI, analyzing how LNPs interact with pro-inflammatory signaling pathways. DL models trained on transcriptomic and proteomic datasets can identify biomarkers associated with inflammation and help to design lipid structures with reduced immune activation^166^, ^167^, ^168^. ML can further refine LNP formulations by iteratively optimizing lipid composition to minimize inflammatory markers such as IL-6, TNF-*α*, and IFN-*γ*, while maintaining their ability to deliver mRNA efficiently^38^^,^^127^^,^^152^. The RF algorithm is highly flexible, robust and interpretable, and excels in processing and analyzing complex multidimensional data^117^. Bae et al.^38^ used the RF algorithm to analyze multiple parameters of lipids associated with the immune response. The results indicated that the immune potency of LNPs was negatively correlated with ester bridge + tail (EB + T) chain length. Furthermore, AI can help to rationalize the selection of anti-inflammatory modifications, such as alternative lipid structures and biodegradable lipids, to reduce undesired immune activation.

Overall, AI-guided assessment and prediction of immune and inflammatory responses of LNPs enable more precise control of immunogenicity. By integrating ML, DL, and multi-omics data analysis, AI provides valuable guidance for analyzing the immune interactions associated with LNPs, facilitating the development of safer and more effective nucleic acid therapies^169^. These advances not only improve the biocompatibility of LNPs, but also pave the way for personalized nanomedicine, allowing for optimizing the therapeutic efficacy based on individual immune profiles.

## 5 Conclusions

The integration of AI into mRNA-LNP delivery systems has ushered in a transformative era, revolutionizing nanoparticle synthesis, drug delivery optimization, and mRNA-based therapeutics^40^^,^^66^. The synergy between ML techniques and nanotechnology has not only enabled the development of more efficient, targeted mRNA therapies but also deepened our understanding of complex nano-bio interactions that were previously difficult to decipher^152^^,^^170^. Among ML approaches, DL models have demonstrated exceptional capacity to process and interpret vast datasets^12^^,^^41^^,^^171^^,^^172^. These models effectively decode intricate nano-bio interactions, uncovering hidden patterns in nanoscale systems. In the realm of nanoparticle synthesis, ML algorithms are accelerating the discovery of novel materials and formulations by identifying key parameters that influence nanoparticle characteristics, including size, surface charge, and stability. This acceleration significantly reduces the trial-and-error approach, providing a faster and more efficient pathway to optimize the nanoparticle design^173^^,^^174^. Furthermore, AI-driven mRNA delivery optimization enhances precise targeting of therapeutic cargos to specific cells or tissues, thereby enhancing treatment function and minimizing off-target effects. By utilizing predictive models, researchers can better anticipate the pharmacokinetics and biodistribution of nanoparticles *in vivo*, ultimately paving the way for more personalized drug delivery strategies tailored to individual patient profiles.

Despite these promising advancements, the integration of AI into mRNA-LNPs remains in its early stages and presents several key challenges. While AI holds transformative potential for accelerating mRNA-LNP design, several critical limitations must be addressed. Firstly, the inherent “black-box” nature of current AI models poses dual challenges: their limited interpretability in feature selection and opaque decision-making processes impede both scientific understanding and clinical approval^175^^,^^176^, although the utility of SHapley Additive exPlanations can partially explain the results of the model. For instance, models predicting organ-targeting of LNPs may inadequately distinguish whether the selectivity originates from lipid composition, particle size dynamics, or transient protein corona interactions, thereby hindering systematic optimization. Secondly, current data ecosystems exhibit inherent limitations. Training datasets are disproportionately skewed toward specific lipid classes (*e.g.*, SM-102-dominated ionizable lipids^139^) and murine preclinical models, introducing systemic biases that compromise generalizability across human tissues and pathophysiological states. Furthermore, the scarcity of standardized *in vivo* datasets correlating nanoparticle physicochemical properties (*e.g.*, p*K*_a_ and PEGylation density) with organ-specific biodistribution profiles critically undermines model robustness. Thirdly, a fundamental disconnect persists between *in silico*/*in vitro* predictions and *in vivo* outcomes. AI models trained on *in vitro* transfection efficiency metrics often fail to account for physiological complexity (*e.g.*, immune surveillance mechanisms, endothelial barrier dynamics, and time-dependent protein corona evolution), resulting in poor predictable accuracy for organ-specific delivery. Emerging hybrid prediction systems that integrate multi-omics data with advanced *in vitro* platforms (organoids, organ-on-a-chip) aim to address this gap by recapitulating tissue-level biological complexity^177^^,^^178^. Finally, even with accurate predictions, translating AI-designed LNPs into clinical therapies faces regulatory uncertainties. Current guidelines lack frameworks for validating “black-box” algorithms, while ethical concerns persist regarding data privacy (*e.g.*, patient-specific mRNA designs) and algorithmic accountability. Collaborative efforts among computational scientists, regulatory agencies, and clinicians are urgently required to establish standardized evaluation protocols.

The success of ML in designing LNPs also faces dual challenges of data quantity and quality. The current datasets in the mRNA-LNP field are usually limited in size and suffer from issues such as parameter dispersion, high dimensionality, and experimental noise. However, obtaining reliable results does not rely solely on data volume, and can also be balanced through some strategies. For small datasets (<100), setting appropriate learning rates and turn counts, the XGboost model can also achieve accurate prediction of multiple response variables (*R*^2^ > 0.95, RMSE<0.91, and MAE<0.63)^127^. Using a RF model and oversampling methods on small datasets can also identify the percentage of LNP transport and dynamic results over 24 h^157^. Constructing physical and chemical descriptors, screening feature variables, optimizing model hyperparameters, and using bootstrap methods to resample and expand the dataset may all overcome the problem of inaccurate prediction results caused by small sample sizes, improving model performance to a certain extent. The larger the amount of data, the wider the feature space can usually be covered, reducing the risk of underfitting and improving generalization ability by decreasing overfitting risk through data diversity. In addition, more data can help the model better separate noise and real signals, capture complex nonlinear relationships, support more complex model structures and accurate parameter optimization, thereby significantly improving the accuracy of predictions. When the data volume reaches a certain level (>100), the quality of the data is also quite significant. In case of merging different batches of data, it is necessary to remove batch effects from the dataset itself, unify variables, and perform preprocessing such as noise filtering.

Looking ahead, AI-guided mRNA-LNP systems show great promise for clinical translation, but also encounter numerous challenges. Although AI has demonstrated significant potential in preclinical optimization, it remains difficult to extrapolate its predictions to the human system due to inter-species differences and the lack of highly relevant human data. In order to enhance the applicability of AI models in the clinic, future research should strengthen data sharing to address the problem of high-quality data shortage. Meanwhile, the feasibility of AI-guided nanomedicine application in clinical practice can be effectively accelerated by integrating a series of diversified and high-quality data, including organoid models derived from patients, pharmacogenomics, transcriptomics, protein interactions, and actual clinical data. In addition, the acceptance of AI-assisted drug design also depends on the transparency, reproducibility, and rigorous experimental validation of its predictive models. Close collaboration among computer scientists, drug design researchers, and clinicians will be critical to overcoming these obstacles. By addressing these challenges, AI will not only accelerate the drug development process but also promote the advancement of a new generation of precise nanomedicine, ultimately improving the patient health.

## Author contributions

Kexin Su: Writing - original draft & editing, Conceptualization, Investigation. Junjie Qiu: Writing - original draft & editing, Conceptualization, Investigation. Tengfei Xu: Conceptualization, Resources, Investigation. Shuai Liu: Writing - review & editing, Conceptualization, Resources, Supervision, Funding acquisition, Investigation. All of the authors have read and approved the final manuscript.

## Conflicts of interest

The authors have no conflicts of interest to declare.
